# The Potential Link Between Abdominal Migraine and Chronic Apical Periodontitis: A Case Report

**DOI:** 10.7759/cureus.78270

**Published:** 2025-01-30

**Authors:** Fuu Sakai, Kana Ozasa, Kohei Shimizu, Noboru Noma

**Affiliations:** 1 Oral Medicine, Nihon University School of Dentistry, Tokyo, JPN; 2 Endodontics, Nihon University School of Dentistry, Tokyo, JPN

**Keywords:** abdominal migraine, apical periodontitis, chronic periodontitis, neurovascular mechanisms, orofacial pain

## Abstract

According to the International Classification of Headache Disorders, Third edition (ICHD-3), abdominal migraine is a diagnosis of exclusion, characterized by recurrent abdominal pain accompanied by nausea, vomiting, anorexia, or pallor. We report a case of a 44-year-old female with abdominal migraine associated with chronic apical periodontitis. Treatment with root canal therapy and periodontal management improved both the oral condition and migraine symptoms. Chronic periodontitis may contribute to migraines via systemic inflammation and calcitonin gene-related peptide (CGRP)-mediated neurovascular mechanisms. This case underscores the importance of dental practitioners identifying and managing periodontal conditions to address potential systemic effects and multifactorial orofacial pain.

## Introduction

Patients may present to dental practices with complaints of migraines, and among these, a subset may suffer from abdominal migraines [1]. These can often be effectively managed with migraine-specific medications. Although abdominal migraine predominantly occurs during childhood, it has also been documented in adults through case studies. The International Headache Society (IHS) has established diagnostic criteria for abdominal migraines [2]. Typically, patients present with recurrent, episodic abdominal pain accompanied by at least two of the following symptoms: loss of appetite, nausea, vomiting, or pallor. This condition is often observed in women with a predisposition to or a history of migraines. Abdominal migraine remains a diagnosis of exclusion, confirmed only after ruling out other potential causes of abdominal pain [3].

Inflammatory mediators, bacteria, and bacterial by-products from periodontitis are known to enter systemic circulation, potentially contributing to migraine development [4-8]. The objective of this case report is to explore the clinical characteristics, diagnostic process, and management strategies for a patient presenting with both abdominal migraine and chronic apical periodontitis. Through this report, we aim to investigate the potential link between these conditions and highlight the importance of interdisciplinary healthcare approaches.

## Case presentation

The authors confirm that they have acquired suitable patient consent forms, whereby the patient has granted permission for the sharing of her images and clinical information in a journal while opting not to disclose her name or initials.

A 44-year-old female patient presented with a history of intermittent pain in the left maxillary region, extending from the nasal ala to the cheek, occurring one to two times daily for approximately three to four years. Initially diagnosed with non-dental tooth pain at her regular dental clinic, she was referred to our university hospital department for further evaluation and treatment. During headache attacks, she experienced associated symptoms such as abdominal pain, nausea, and vomiting but did not experience any aura symptoms, photophobia, or phonophobia. Daily, she reported a baseline pain intensity of 4/10 on the Visual Analog Scale (VAS) localized around the inferior margin of the zygomatic arch, which escalated to 10/10 during headache episodes. The patient was ruled out for irritable bowel syndrome (IBS) by the gastroenterologist. She was diagnosed with migraines at a headache clinic. While loxoprofen sodium provided no relief, the use of triptans and anti-calcitonin gene-related peptide (CGRP) antibodies reduced the intensity of her migraine attacks. The patient did not experience phenomena such as flashes of light, zigzag lines, or scotoma (partial vision loss), nor was there any scalp allodynia. Additionally, there was no referred pain to the oral cavity during the headache attacks.

Radiographic findings

**Figure 1:**
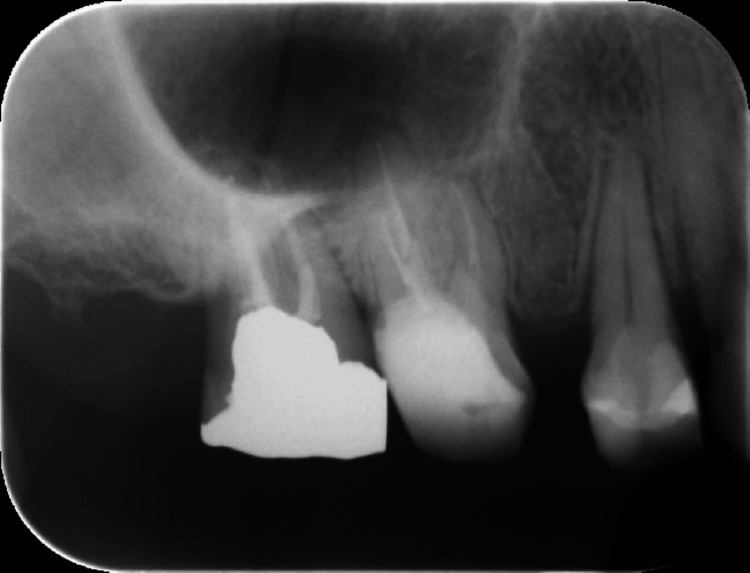
Periapical radiograph Periapical radiographs revealed a slight radiolucent area at the palatal root apex of the upper right first molar.

**Figure 2:**
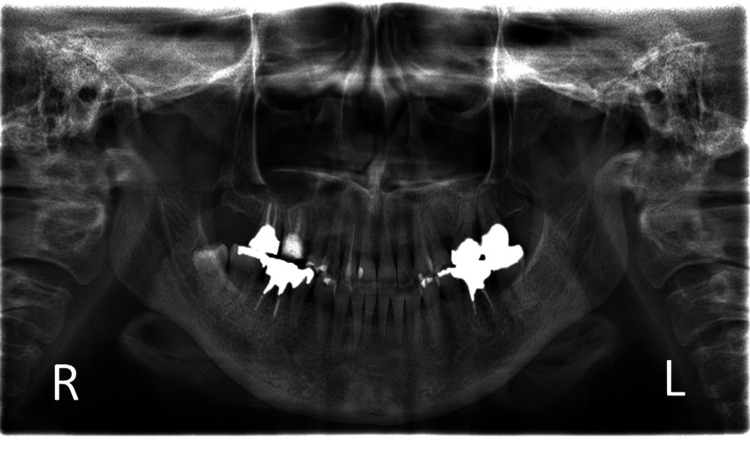
Panoramic radiographs Panoramic radiographs revealed a slight radiolucent area at the palatal root apex of the upper right first molar, but no alveolar bone resorption or other abnormal findings.

Neurological findings were normal, as was the temporomandibular joint. Muscle palpation elicited severe tenderness in the right temporalis muscle and the left masseter muscle, but this did not reproduce the chief complaint. Intraoral examination revealed generalized moderate inflammation of the gingiva. Additionally, percussion sensitivity was noted in the maxillary right first molar. While panoramic radiographs showed no abnormalities in the dentition or maxillary sinuses, periapical radiographs revealed a slight radiolucent area at the root apices of the upper right first and second molars (Figures 1, 2).

The pain was determined to be odontogenic. The potential for the odontogenic pain and periodontal disease to exacerbate her migraines was explained. Root canal treatment and periodontal therapy were then completed for the right upper first molar by an endodontist using a dental operating microscope. Three months after the treatment, the percussion pain had decreased, periodontal condition had improved, and the frequency of migraine attacks had decreased accordingly.

## Discussion

According to the ICHD-3, migraines are characterized by recurrent headaches of moderate to severe intensity lasting 4 to 72 hours, with a pulsating quality, unilateral presentation, and worsening with routine physical activity, accompanied by nausea and either phonophobia, photophobia, or both [2]. In abdominal migraines, abdominal pain is often not localized to the central abdomen and is accompanied by symptoms such as nausea and vomiting, which are typical of traditional migraines.

The case reports of abdominal migraine have been limited to 12 cases, including our own, and to our knowledge, this is the first report of abdominal migraine associated with odontogenic tooth pain [1, 6, 9-14] (Table 1). 

**Table 1:** Case reports and treatment details for abdominal migraine The present case and 12 other cases of abdominal migraine have been recognized to date. The table outlines the duration of treatment and treatment methods.

Author	Age	Sex	Disease duration	Family history of migraine	Treatment (abortive medication)	Treatment (preventive medication)
Woodruff et al., 2013 [1]	32	Female	5-6 years	Positive	-	Topiramate
Roberts et al., 2012 [6]	48	Female	2 years	Positive	Rizatriptan	Topiramate
	24	Female	5 years	Negative	-	Topiramate
Cervellin et al., 2015 [9]	30	Female	15 years	Negative	Ketoprofen	-
					Metoclopramide	
Hamed, 2010 [10]	20	Male	10 years	Positive	Eletriptan	Valproate
d’Onofrio et al., 2006 [11]	23	Female	7 years	Positive	-	Rimegepant
Newman et al., 2008 [12]	22	Male	4 years	Positive	Sumatriptan	Topiramate,Verapami
Evans et al., 2013 [13]	52	Male	3 years	Negative	Eletriptan	-
					Prochlorperazine	
	56	Male	16 months	Negative	-	Propranolol, Amitriptyline
						Nebivolol, Venlafaxine
	27	Female	3 years	Positive	Eletriptan	Topiramate
Kunishi et al., 2016 [14]	52	Female	1 month	Positive	Loxoprofen	Migexis
Our case	44	Female	3-4 years	Negative	Loxoprofen	Anti-CGRP antibody
					Sumatriptan	

The age range of cases is 20 to 56 years, with an average age of 36.3 years. The age group distribution is 33.3% of cases in the 20-29 years range, 16.7% in the 30-39 years range, 16.7% in the 40-49 years range, and 33.3% in the 50 years and above range. The gender distribution is 66.7% female and 33.3% male. Reported disease durations range from one month to 15 years and an average of 5.5 years, with 8.3% of patients having a duration of less than one year, 58.3% having one to five years, and 33.3% having more than five years. Fifty percent of patients had a family history of migraine. Triptans were the most commonly used acute medication (50%), while topiramate was the most frequent preventive treatment. Anti-CGRP antibodies were used in one case (our own)．

In our case, proton pump inhibitors were ineffective when abdominal pain occurred, but triptans were effective, leading to a diagnosis of abdominal migraine by a headache specialist. Effective migraine medication could be considered a key factor for the differential diagnosis. The anti-CGRP antibodies are new preventive medications with the potential to help patients who have not responded adequately to traditional treatments such as triptans or nonsteroidal anti-inflammatory drugs (NSAIDs).

Chronic periodontitis is a persistent inflammatory disease that affects the tooth-supporting structures, including the gingiva, periodontal ligament, and alveolar bone [15]. It is primarily initiated by the accumulation of bacterial biofilm on dental surfaces, which elicits a host immune response. Key clinical features include gingival erythema, edema, bleeding on probing, and the formation of deep periodontal pockets. If left untreated, the condition can lead to progressive attachment loss, tooth mobility, and ultimately tooth loss. Recent studies have demonstrated that it contributes to systemic inflammatory burden, influencing various systemic conditions, including atherosclerosis (cardiovascular and cerebrovascular diseases), rheumatoid arthritis, pregnancy complications, chronic kidney disease, and Alzheimer's disease [16].

Apical periodontitis has also been suggested to trigger migraines and potentially contribute to their chronification [17]. Leira et al. reported that migraine patients diagnosed with periodontitis exhibited significantly higher levels of gingival inflammation compared to migraine patients without periodontitis [18].

Numerous studies show that patients with periodontal disease have elevated levels of systemic inflammatory mediators compared to healthy controls, including C-reactive protein (CRP), interleukin 1 (IL-1), interleukin 6 (IL-6), and tumor necrosis factor-α (TNF-α) [17]. This pattern mirrors the inflammatory mediator expression found in migraine patients. These cytokines are associated with vascular dysfunction and are pro-inflammatory. A study by Leira et al. identified leptin and procalcitonin as promising biomarkers elevated in both chronic periodontitis and migraine patients [18].

On the other hand, vasodilation and enhanced vascular permeability are key mechanisms in neurogenic inflammation during migraines, involving neuropeptides such as substance P (SP), neurokinin A (NKA), and calcitonin gene-related peptide (CGRP). These neuropeptides modulate migraine molecular events, with CGRP being a critical mediator. Serum CGRP levels fluctuate with migraine severity, rising during attacks. The pathophysiology of both inflammatory periodontitis and migraines is closely linked to CGRP, highlighting its role in both conditions [4]. This suggests a shared neurovascular mechanism between periodontal inflammation and migraine occurrence.

**Figure 3:**
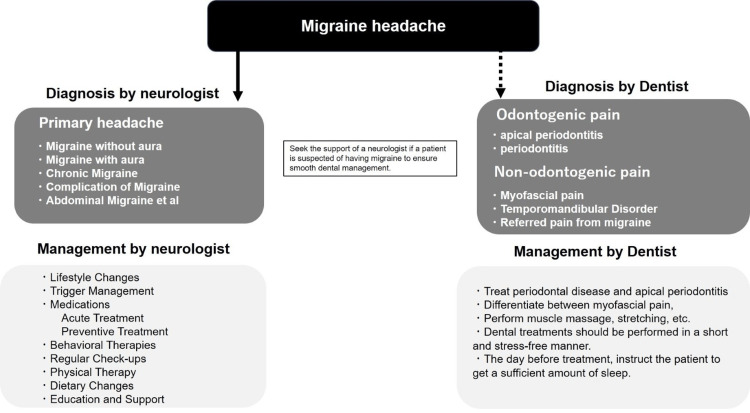
Flowchart for the diagnosis and management of migraine patients involving neurologists and dentists Image credits: NN

In this case, the root canal treatment and periodontal therapy led to a reduction in migraine symptoms. We used fremanezumab, a monoclonal antibody targeting CGRP. This treatment has been shown to reduce the frequency and severity of migraine attacks by inhibiting the action of CGRP. Given that CGRP plays a crucial role in neurovascular responses during migraines, fremanezumab is expected to be effective based on this mechanism, leading to clinical improvement in migraine patients. Periodontal disease is considered a low-grade inflammatory condition that may stimulate vasoactive mediators, potentially contributing to the onset of migraines. A flowchart of diagnosis and treatment for migraine patients is proposed in Figure 3. Dentists need to be able to differentiate orofacial pain due to odontogenic and non-odontogenic causes (e.g., migraine). If a patient is suspected of having migraines, the dentist should refer the patient to a neurologist for a definitive diagnosis and to ensure smooth dental management. After a migraine diagnosis from a neurologist, treatment for apical periodontitis and/or periodontal disease, whichever is present, can be initiated. To exclude non-odontogenic factors that may trigger migraines, temporomandibular joint (TMJ) and masticatory muscles should be assessed. If a symptomatic temporomandibular disorder TMD is found to be present, stretching and massaging should be done as needed, and dental treatments should be conducted in a short and stress-free manner.

## Conclusions

In conclusion, this case highlights the importance for dentists to differentiate between odontogenic and non-odontogenic orofacial pain, such as migraines. The potential association between migraines and periodontitis underscores the need for dental professionals to consider periodontal conditions as a contributing factor when diagnosing orofacial pain. This study's findings suggest that treating periodontal disease may have a positive impact on migraine symptoms, emphasizing the role of oral health in overall patient well-being. Future research should also investigate the long-term effects of periodontal treatment on migraine prevention and explore other potential links between oral health and systemic conditions.
